# Supplementary figures and images for: An optimized electrotransformation protocol for Lactobacillus jensenii
Source: PLoS One. 2023 Feb 17;18(2):e0280935. doi: 10.1371/journal.pone.0280935 (PMC9937494; doi:10.1371/journal.pone.0280935)

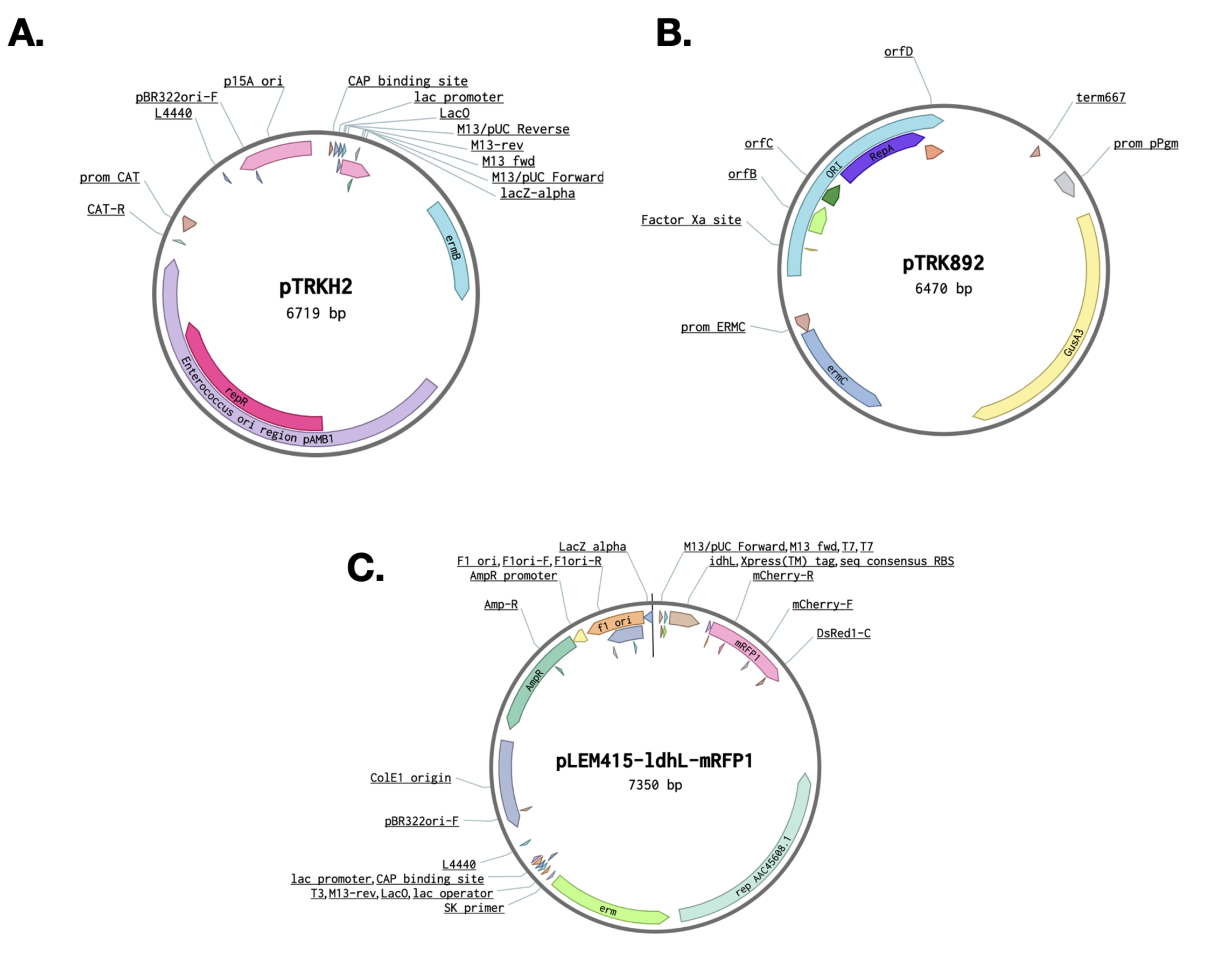

Supplement: S1 Fig — A. pTRKH2. B. pTRK892. C. pLEM415-ldhl-mRFP1. (TIFF) [file pone.0280935.s001.tiff]

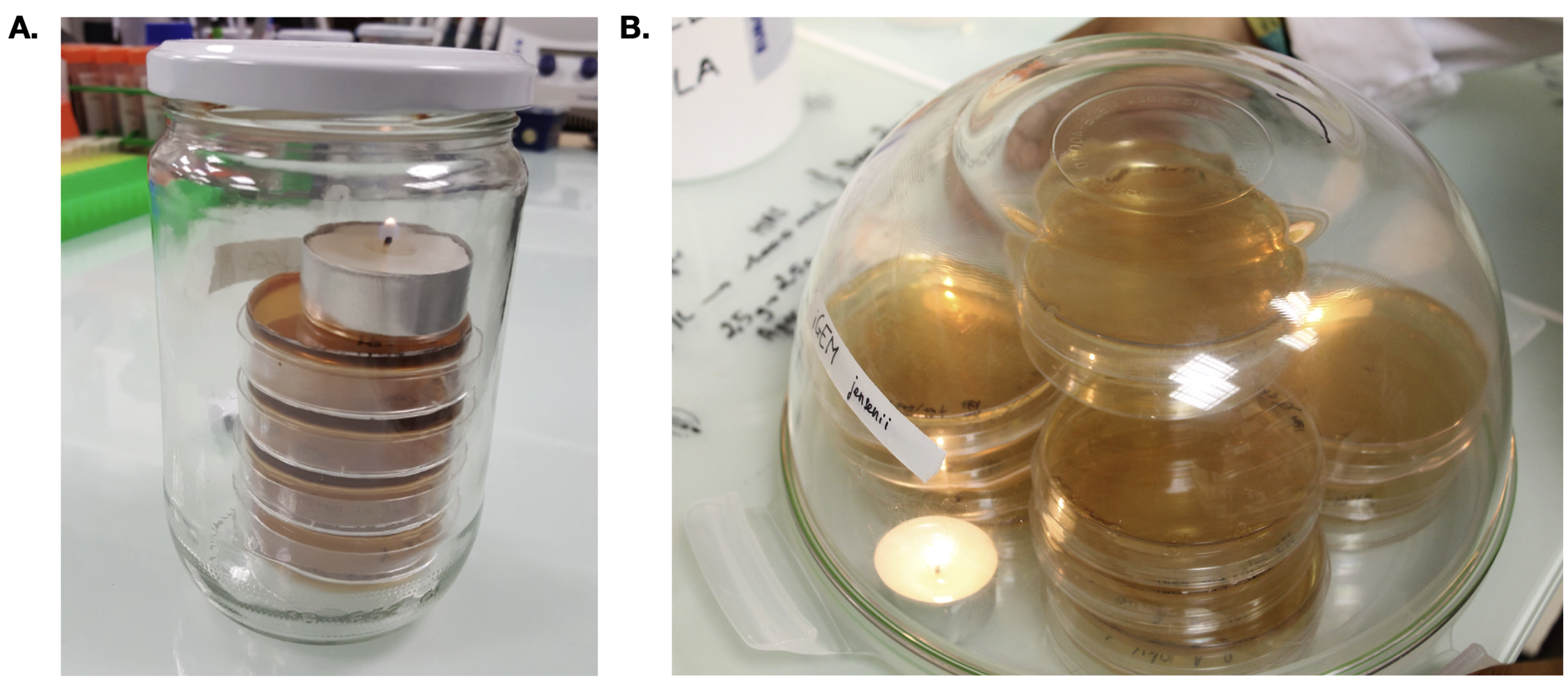

Supplement: S2 Fig — A. Small plates (60mm diameter) are placed in a hermetically closed jar with a burning candle. B. 100mm plates incubated in an airtight-closing bowl with candles. (TIFF) [file pone.0280935.s002.tiff]
