# Supplementary material for: An optimized electrotransformation protocol for Lactobacillus jensenii
Source: PLoS One. 2023 Feb 17;18(2):e0280935. doi: 10.1371/journal.pone.0280935 (PMC9937494; doi:10.1371/journal.pone.0280935)
Supplement: S1 Table — (DOCX) [file pone.0280935.s003.docx]

| **Primers** | **Sequence** | **Amplicon size** |
| --- | --- | --- |
| pFOR pTRKH2 | tctgacgctcaaatcagtgg | 500 bp |
| pREV pTRKH2 | gttttggtctgcgcgtaatc |  |
| pFOR 16S *L. jensenii* | ggcgtaagctgtcgtaaagg | 2000 bp |
| pREV 16S *L. jensenii* | tcatctgtcccaccttaggc |  |
