# Supplementary material for: An optimized electrotransformation protocol for Lactobacillus jensenii
Source: PLoS One. 2023 Feb 17;18(2):e0280935. doi: 10.1371/journal.pone.0280935 (PMC9937494; doi:10.1371/journal.pone.0280935)
Supplement: S2 Table — (DOCX) [file pone.0280935.s004.docx]

| Fig2A Starting protocol : | Triplicat 1 | Triplicat 2 | Triplicat 3 |
| --- | --- | --- | --- |
| Luchansky et al protocol | 83 | 32 | 34 |
|  | 68 | 39 | 22 |
|  | 61 | 48 | 40 |
| Berthier et al protocol | 0 | 5 | 15 |
|  | 12 | 5 | 0 |
|  | 10 | 9 | 6 |
|  |  |  |  |
| Fig2B Buffer strength | Triplicat 1 | Triplicat 2 | Triplicat 3 |
| 1X | 50 | 160 | 80 |
|  | 30 | 30 | 40 |
|  | 20 | 10 | 30 |
| 2X | 400 | 230 | 180 |
|  | 210 | 40 | 200 |
|  | 140 | 100 | 150 |
| 3X | 350 | 460 | 500 |
|  | 240 | 780 | 430 |
|  | 440 | 450 | 640 |
|  |  |  |  |
| Fig2C Glycine | Triplicat 1 | Triplicat 2 | Triplicat 3 |
| 0 | 350 | 460 | 500 |
|  | 240 | 780 | 430 |
|  | 440 | 450 | 640 |
| 0.5 | 350 | 440 | 490 |
|  | 150 | 460 | 640 |
|  | 460 | 790 | 430 |
| 1.0 | 230 | 330 | 320 |
|  | 0 | 350 | 540 |
|  | 210 | 450 | 630 |
| 1.5 | 410 | 520 | 670 |
|  | 370 | 580 | 680 |
|  | 690 | 650 | 550 |
| 2.0 | 430 | 530 | 730 |
|  | 910 | 830 | 940 |
|  | 820 | 750 | 790 |
|  |  |  |  |
| Fig3A Cuvette size | Triplicat 1 | Triplicat 2 | Triplicat 3 |
| 0.1 | 430 | 530 | 730 |
|  | 910 | 830 | 940 |
|  | 820 | 750 | 790 |
| 0.2 | 300 | 1240 | 1500 |
|  | 1400 | 1880 | 1300 |
|  | 2020 | 1510 | 1340 |
|  |  |  |  |
| Fig2B DNA quantity | Triplicat 1 | Triplicat 2 | Triplicat 3 |
| 0 | 0 | 0 | 0 |
|  | 0 | 0 | 0 |
|  | 0 | 0 | 0 |
| 0.5 | 10 | 33 | 5 |
|  | 34 | 23 | 23 |
|  | 0 | 50 | 30 |
| 1.0 | 300 | 1240 | 1500 |
|  | 1400 | 1880 | 1300 |
|  | 2020 | 1510 | 1340 |
| 2.0 | 500 | 1300 | 1390 |
|  | 1650 | 810 | 1020 |
|  | 1420 | 1830 | 1800 |
| 4.0 | 1950 | 1750 | 2070 |
|  | 1620 | 1530 | 1200 |
|  | 1920 | 1500 | 2100 |
|  |  |  |  |
| Fig3C Electric field | Triplicat 1 | Triplicat 2 | Triplicat 3 |
| 1 | 0 | 0 | 0 |
|  | 0 | 10 | 0 |
|  | 0 | 1 | 0 |
| 2,5 | 300 | 200 | 330 |
|  | 250 | 100 | 180 |
|  | 190 | 180 | 330 |
| 4,5 | 400 | 440 | 390 |
|  | 1210 | 830 | 740 |
|  | 820 | 840 | 650 |
| 6,5 | 300 | 1240 | 1500 |
|  | 1400 | 1880 | 1300 |
|  | 2020 | 1510 | 1340 |
| 12,5 | 2500 | 2100 | 2510 |
|  | 1540 | Arc | 2360 |
|  | 2120 | 2300 | 2700 |
|  |  |  |  |
| Fig4A Plasmids | Triplicat 1 | Triplicat 2 | Triplicat 3 |
| pTRKH 2 | 2500 | 2100 | 2510 |
|  | 1540 | Arc | 2360 |
|  | 2120 | 2300 | 2700 |
| pTRK 892 | 5000 | 1000 | 6020 |
|  | 4300 | 2210 | 3500 |
|  | 3210 | 4500 | 1960 |
| pLEM415 | 42 | 0 | 31 |
|  | 20 | 18 | 82 |
|  | 29 | 12 | 21 |
|  |  |  |  |
| Fig4C Strains | Triplicat 1 | Triplicat 2 | Triplicat 3 |
| L.jensenii ATCC2256 NO | 83 | 32 | 34 |
|  | 68 | 39 | 22 |
|  | 61 | 48 | 40 |
| *L.jensenii ATCC2256 O* | 2500 | 2100 | 2510 |
|  | 1540 | Arc | 2360 |
|  | 2120 | 2300 | 2700 |
| Strain #1 NO | 0 | 0 | 0 |
|  | 3 | 0 | 0 |
|  | 0 | 2 | 0 |
| Strain #1 O | 12 | 24 | 14 |
|  | 32 | 7 | 18 |
|  | 23 | 31 | 25 |
| Strain #2 NO | 10 | 0 | 2 |
|  | 2 | 2 | 5 |
|  | 4 | 5 | 3 |
| Strain #2 O | 35 | 102 | 105 |
|  | 46 | 86 | 121 |
|  | 97 | 94 | 93 |
| Strain #3 NO | 3 | 8 | 4 |
|  | 1 | 6 | 0 |
|  | 2 | 3 | 2 |
| Strain #3 O | 130 | 10 | 230 |
|  | 140 | 61 | 110 |
|  | 121 | 40 | 89 |
