# Supplementary material for: An optimized electrotransformation protocol for Lactobacillus jensenii
Source: PLoS One. 2023 Feb 17;18(2):e0280935. doi: 10.1371/journal.pone.0280935 (PMC9937494; doi:10.1371/journal.pone.0280935)
Supplement: S3 Table — (DOCX) [file pone.0280935.s005.docx]

| **Figure** | **Level 1** | **Level 2** | **Test** | **p-value** |  |
| --- | --- | --- | --- | --- | --- |
| **2A** | Protocol Luchansky | Protocol Berthier | Wilcoxon | 4.07e-04 | 4.11e-05 |
| **2B** | Buffer 1X | Buffer 2X | Wilcoxon | 3.02e-03 | 6.29e-03 |
| **2B** | Buffer 1X | Buffer 3X | Wilcoxon | 4.01e-04 | 4.11e-05 |
| **2B** | Buffer 2X | Buffer 3X | Wilcoxon | 1.65e-04 | 7.40e-04 |
| **2C** | Glycine 0 | Glycine 0.5 | Wilcoxon | 1.00e+00 | 1.00e+00 |
| **2C** | Glycine 0 | Glycine 1 | Wilcoxon | 1.12e-01 | 3.52e-01 |
| **2C** | Glycine 0 | Glycine 1.5 | Wilcoxon | 1.61e-01 | 1.26e-01 |
| **2C** | Glycine 0 | Glycine 2 | Wilcoxon | 9.15e-03 | 3.36e-02 |
| **2C** | Glycine 0.5 | Glycine 1 | Wilcoxon | 1.45e-01 | 3.25e-01 |
| **2C** | Glycine 0.5 | Glycine 1.5 | Wilcoxon | 1.57e-01 | 1.10e-01 |
| **2C** | Glycine 0.5 | Glycine 2 | Wilcoxon | 1.03e-02 | 2.90e-02 |
| **2C** | Glycine 1 | Glycine 1.5 | Wilcoxon | 5.64e-03 | 3.36e-02 |
| **2C** | Glycine 1 | Glycine 2 | Wilcoxon | 7.82e-04 | 6.29e-03 |
| **2C** | Glycine 1.5 | Glycine 2 | Wilcoxon | 1.42e-02 | 6.29e-03 |
| **3A** | Cuvette 0.1 | Cuvette 0.2 | Wilcoxon | 3.99e-03 | 7.40e-04 |
| **3B** | DNA quantity 0.25 | DNA quantity 0.5 | Wilcoxon | 4.57e-03 | 6.99e-03 |
| **3B** | DNA quantity 0.25 | DNA quantity 1 | Wilcoxon | 4.10e-04 | 1.55e-04 |
| **3B** | DNA quantity 0.25 | DNA quantity 2 | Wilcoxon | 4.05e-04 | 1.55e-04 |
| **3B** | DNA quantity 0.25 | DNA quantity 4 | Wilcoxon | 1.73e-03 | 2.02e-03 |
| **3B** | DNA quantity 0.5 | DNA quantity 1 | Wilcoxon | 8.55e-03 | 1.86e-02 |
| **3B** | DNA quantity 0.5 | DNA quantity 2 | Wilcoxon | 9.23e-04 | 1.55e-04 |
| **3B** | DNA quantity 0.5 | DNA quantity 4 | Wilcoxon | 8.36e-03 | 4.04e-03 |
| **3B** | DNA quantity 1 | DNA quantity 2 | Wilcoxon | 8.75e-01 | 6.22e-01 |
| **3B** | DNA quantity 1 | DNA quantity 4 | Wilcoxon | 6.83e-01 | 8.36e-01 |
| **3B** | DNA quantity 2 | DNA quantity 4 | Wilcoxon | 2.69e-01 | 4.69e-01 |
| **3C** | KV/cm 1 | KV/cm 2.5 | Wilcoxon | 2.70e-04 | 4.11e-05 |
| **3C** | KV/cm 1 | KV/cm 4.5 | Wilcoxon | 2.74e-04 | 4.11e-05 |
| **3C** | KV/cm 1 | KV/cm 6.5 | Wilcoxon | 2.74e-04 | 4.11e-05 |
| **3C** | KV/cm 1 | KV/cm 12.5 | Wilcoxon | 4.01e-04 | 8.23e-05 |
| **3C** | KV/cm 2.5 | KV/cm 4.5 | Wilcoxon | 4.07e-04 | 4.11e-05 |
| **3C** | KV/cm 2.5 | KV/cm 6.5 | Wilcoxon | 9.12e-04 | 7.40e-04 |
| **3C** | KV/cm 2.5 | KV/cm 12.5 | Wilcoxon | 6.26e-04 | 4.11e-05 |
| **3C** | KV/cm 4.5 | KV/cm 6.5 | Wilcoxon | 3.99e-03 | 7.40e-04 |
| **3C** | KV/cm 4.5 | KV/cm 12.5 | Wilcoxon | 8.23e-05 | 8.23e-05 |
| **3C** | KV/cm 6.5 | KV/cm 12.5 | Wilcoxon | 3.29e-04 | 1.40e-03 |
| **4A** | pTRKH2 | pTRK892 | Wilcoxon | 1.39e-01 | 2.02e-02 |
| **4A** | pTRKH2 | pLEM415 | Wilcoxon | 8.23e-05 | 8.23e-05 |
| **4A** | pTRK892 | pLEM415 | Wilcoxon | 4.11e-05 | 4.11e-05 |
| **4C** | L. jensenii ATCC2256 Non-Optimized | L. jensenii ATCC2256 Optimized | Wilcoxon | 8.23e-05 | 8.23e-05 |
| **4C** | L. jensenii ATCC2256 Non-Optimized | Strain #1 Non-Optimized | Wilcoxon | 2.74e-04 | 4.11e-05 |
| **4C** | L. jensenii ATCC2256 Non-Optimized | Strain #1 Optimized | Wilcoxon | 2.30e-03 | 6.29e-03 |
| **4C** | L. jensenii ATCC2256 Non-Optimized | Strain #2 Non-Optimized | Wilcoxon | 3.98e-04 | 4.11e-05 |
| **4C** | L. jensenii ATCC2256 Non-Optimized | Strain #2 Optimized | Wilcoxon | 5.64e-03 | 6.29e-03 |
| **4C** | L. jensenii ATCC2256 Non-Optimized | Strain #3 Non-Optimized | Wilcoxon | 4.07e-04 | 4.11e-05 |
| **4C** | L. jensenii ATCC2256 Non-Optimized | Strain #3 Optimized | Wilcoxon | 3.39e-02 | 2.90e-02 |
| **4C** | L. jensenii ATCC2256 Optimized | Strain #1 Non-Optimized | Wilcoxon | 4.01e-04 | 8.23e-05 |
| **4C** | L. jensenii ATCC2256 Optimized | Strain #1 Optimized | Wilcoxon | 8.23e-05 | 8.23e-05 |
| **4C** | L. jensenii ATCC2256 Optimized | Strain #2 Non-Optimized | Wilcoxon | 6.11e-04 | 8.23e-05 |
| **4C** | L. jensenii ATCC2256 Optimized | Strain #2 Optimized | Wilcoxon | 8.23e-05 | 8.23e-05 |
| **4C** | L. jensenii ATCC2256 Optimized | Strain #3 Non-Optimized | Wilcoxon | 6.26e-04 | 8.23e-05 |
| **4C** | L. jensenii ATCC2256 Optimized | Strain #3 Optimized | Wilcoxon | 8.23e-05 | 8.23e-05 |
| **4C** | Strain #1 Non-Optimized | Strain #1 Optimized | Wilcoxon | 2.74e-04 | 4.11e-05 |
| **4C** | Strain #1 Non-Optimized | Strain #2 Non-Optimized | Wilcoxon | 6.06e-03 | 2.21e-02 |
| **4C** | Strain #1 Non-Optimized | Strain #2 Optimized | Wilcoxon | 2.74e-04 | 4.11e-05 |
| **4C** | Strain #1 Non-Optimized | Strain #3 Non-Optimized | Wilcoxon | 8.16e-03 | 2.21e-02 |
| **4C** | Strain #1 Non-Optimized | Strain #3 Optimized | Wilcoxon | 2.74e-04 | 4.11e-05 |
| **4C** | Strain #1 Optimized | Strain #2 Non-Optimized | Wilcoxon | 5.55e-04 | 7.40e-04 |
| **4C** | Strain #1 Optimized | Strain #2 Optimized | Wilcoxon | 4.11e-05 | 4.11e-05 |
| **4C** | Strain #1 Optimized | Strain #3 Non-Optimized | Wilcoxon | 5.66e-04 | 7.40e-04 |
| **4C** | Strain #1 Optimized | Strain #3 Optimized | Wilcoxon | 2.76e-03 | 7.40e-04 |
| **4C** | Strain #2 Non-Optimized | Strain #2 Optimized | Wilcoxon | 3.98e-04 | 4.11e-05 |
| **4C** | Strain #2 Non-Optimized | Strain #3 Non-Optimized | Wilcoxon | 7.88e-01 | 1.00e+00 |
| **4C** | Strain #2 Non-Optimized | Strain #3 Optimized | Wilcoxon | 4.67e-04 | 7.40e-04 |
| **4C** | Strain #2 Optimized | Strain #3 Non-Optimized | Wilcoxon | 4.07e-04 | 4.11e-05 |
| **4C** | Strain #2 Optimized | Strain #3 Optimized | Wilcoxon | 4.53e-01 | 3.29e-01 |
| **4C** | Strain #3 Non-Optimized | Strain #3 Optimized | Wilcoxon | 4.07e-04 | 4.11e-05 |
