# Supplementary material for: An optimized electrotransformation protocol for Lactobacillus jensenii
Source: PLoS One. 2023 Feb 17;18(2):e0280935. doi: 10.1371/journal.pone.0280935 (PMC9937494; doi:10.1371/journal.pone.0280935)
Supplement: S1 Protocol — (DOCX) [file pone.0280935.s006.docx]

| **1. Buffer preparation** | | |
| --- | --- | --- |
| SMEB 3X (Sucrose Magnesium Electroporation Buffer 1X: 298mM Sucrose, 1mM MgCl2) | H20 milliQ | 100 mL qsp |
|  | Sucrose | 32.5 g |
|  | MgCl2 | 0.06 g |
| Add 50 mL of water first before adding sucrose and MgCl2 and mix it carefully until sucrose completely melts. Storage at 4°C at least all night before use and conservation in the fridge at 4°C for three to four months. | | |

| **2. Growth conditions** | | |
| --- | --- | --- |
| Day -1 | Overnight incubation of a pre-culture of *L. jensenii* WT from glycerol stock in 50 mL of MRS liquid media without antibiotics at 37°C without shaking in the anaerobic jar with GasPak system. | **16 hours** |
| Day 0 | Inoculate 2.0 mL from overnight culture in 100 mL of MRS liquid media supplemented with 2% glycine. | **4-5 hours** |

| **3. Competent cells preparation** | | |
| --- | --- | --- |
| *NB: For 100 mL of culture, 70 mL of cold SMEB 3X buffer is required to prepare the competent cells* | | |
| Step 1. | Separate the 100 mL culture in two 50 mL falcon tubes in sterile conditions | |
| Step 2. | Centrifugation at 14°C, 5000 g for 5 minutes | |
| Step 3. | Threw away supernatant and resuspend each the pellet in 15 mL cold SMEB 3X | |
| *Do everything in ice from this point* | | |
| Step 4. | Centrifugation at 5°C, 5000 g for 5 minutes (wash step) | **Repeat 3 times** |
| Step 5. | Threw supernatant, add 15 mL of SMEB 3X and resuspend the pellet. |  |
| *At the last centrifugation step don’t resuspend the pellet (concentration step)* | | |
| Step 6. | After throwing the last supernatant resuspend each pellet in 500 μL of SMEB 3X | |
| Step 7. | Aliquot it in 200 μL samples in cold eppendorf tubes and store a -80°C (no need of flash freeze) | |
| *Cells must be used until one hour or stored at -80° no more than 4 months* | | |

| **4. Electroporation procedure** | |
| --- | --- |
| *Put recovery media (MRS or MRS MgCl2 supplemented) at 37°C 2 hours before the electroporation. Be sure you already have MRS-ERY 0.5 μg plate in stock.* | |
| Step 1. | Thaw competent *L. jensenii* competent 200 μL cells aliquots on ice for 20 minutes. |
| Step 2. | Add at least 1.0 μg of DNA (up to 5.0 μL) and let it rest for between 5-10 minutes with DNA prior to electroporation. |
| Step 3. | At the same time put the electroporation cuvette 0.2 cm on ice, they must be cold. |
| *Electroporation cuvettes can also be placed in the fridge at 4°C before the electroporation.* | |
| Step 4. | Put the 200 μl of cell+DNA in cold electroporation cuvette (0.2 cm), carefully dry the cuvette with a tissue, and electroporate using Gene Pulser II apparatus. |
| *Parameters: 25 μF of capacitance, 400 Ohm resistance and 12.5kV/cm (time constant must be around 8-10.5ms, if not redo the electroporation on a new sample with less DNA volume).* | |
| Step 6. | Immediately resuspend the cells with 800 μL of recovery media and put the resuspension in a culture tube. |
| Step 7. | Incubate 2-3 hours at 37°C in anaerobic jar with gaspak without agitation. |
| Step 8. | Plate 100 μL (or for low transformable plasmid the total fraction concentrated in 100uL after centrifugation at 4000g for 6 min) of each culture on MRS agar petri dish with appropriate antibiotic using 0.4 cm glass beads. Once plated, put the plate in the anaerobic jar with gaspak and incubate at 37°C without shaking for 48 hours. |
| *After 48 hours of incubation, colonies can be counted and used for further applications.* | |
