# Supplementary material for: An optimized electrotransformation protocol for Lactobacillus jensenii
Source: PLoS One. 2023 Feb 17;18(2):e0280935. doi: 10.1371/journal.pone.0280935 (PMC9937494; doi:10.1371/journal.pone.0280935)
Supplement: Attachment — Submitted filename: response to reviewers.docx [file pone.0280935.s007.docx]

Reviewer #1:

This is well-conducted research and the paper is, for the most part, clearly written. I recommend publication with very minor corrections.

We thank the reviewer for his positive assessment. We have corrected all minor points listed below in the revised version of the manuscript.

One thing that must be corrected is a paragraph in the Discussion where the presence of the ermB and ermC genes in the vectors is discussed (page 14). Two sentences in the paragraph are inconsistent with each other concerning which gene is on pTRKH2 and which gene is on the other vector being discussed.

Minor issues:

Key words: Why are ‘Acid Bacteria’ capitalized?

Abstract: Line 9 should ‘strain’ be ‘species’?

Abstract: Line 12 ‘cuvettes type’ should be ‘cuvette type’

Introduction (page 3): ‘bio engineered’ should be ‘bioengineered’

Introduction (last paragraph, six lines from end): ‘species optimized’ should be ‘species and optimized’
Figure 1B ‘additivs’ should be ‘additives’ and ‘vectors selection’ should be ‘vector selection’

Figure 1C why is ‘Cancer’ capitalized?

Figure 1 legend: ‘therapeutics’ should be ‘therapeutic’

Materials and media: third line in first paragraph: ‘Systems) ,’ remove unneeded blank space after the )

Materials and media: line 5 in first paragraph: why is Peptone capitalized?
Materials and media: in the Competent Cells Preparation section, line 3: ‘mL MRS fresh MRS’

should be ’mL fresh MRS’

Results: line 7 in first paragraph: ‘Addgene.’ Delete the period.

The word ‘Greedy’ is used twice in the paper but do you mean ‘Speedy’?
Table 1 legend: ‘abbreviation :’ should be ‘abbreviation:’ without the extra blank space

Table 1 ‘unknow’ should be ‘unknown’

Discussion: Should ‘Bacteroides thetaiotamicron’ be in italics?

Supplemental Figure 1 legend: panel D is mislabeled as a second panel C in the text.

Supplemental Figure 2 legend: Should the last sentence end with a period?

Supplemental Table 1: Should ‘primers’ be ‘primer’?

Supplemental Description of Electroporation protocol:

Step 1: Should ‘threw’ be ‘thaw’?

Step 4: Should ‘a tissues’ be ‘a tissue’? Should ‘electropore’ be ‘electroporate’?

The Time Constant Parameter: here you recommend 9-10 ms but the manuscript says 8-10.5 ms “(electroporation time constant must be in 8-10.5 ms range to show good efficiency)”
Step 8 mentions glass beads, but what size beads and is their use required? I don’t recall seeing them mentioned earlier in the paper.

Reviewer #2:

The title itself “An Optimized Electrotransformation Protocol For Lactobacillus Jensenii“ is misleading, because a single strain of the species has been tested and, considering the variability commonly observed among different strains of the same species, it is hard to assert that the described protocol may be used for the whole L.jensenii species.

This is indeed a common problem and we thank the reviewer for this comment. The same issue is also raised by reviewer 3. To answer this, we have obtained 3 independent *L. jensenii* from a bank established in a local hospital (Arnaud de Villeneuve UMR IRD224-CNRS5290-UM). We tested our protocol on these additional strains and added the results in Figure 4. We found that the protocol does permit to increase the transformability of all strain tested relative to the original procedure, though to various extent (10 to 40-fold increase).

I cannot see the originality of the work, as none of the materials have been generated during this study (both the host and the plasmids have been purchased from public sources) and the setup of the protocol has followed the strategies already described Luchansky and by Berthier in the cited references.

We must agree that this is not a tremendously original work. However, we did combined parameters identified from different sources (Luchansky et al., 1988; Luchansky et al., 1989; Chang et al., 1989; Berthier et al., 1996). In doing so, we believe that we contributed some clarity as to what procedure can be followed for other endeavors of this type. We also think our results on *L. jensenii* can be valuable to others.

Even the transformation efficiency obtained is not better than that reported by the cited authors respectively for L. acidophilus and L.sakei (but in those papers multiple strain for each species were tested).

Transformation efficiency can vary tremendously between strains and species. Some species transform well using standardized procedures, other are well-studied and suitable protocols have already been published. We found *L. Jensenii* to be particularly difficult to transform and experienced more difficulties than expected in optimizing the efficiency reported in this manuscript. That we identified a protocol yielding effixiencies on par with more amenable species is no small feat in our opinion. We very much hope that our data will promote the use of *L. Jensinii* to complement other more amenable strain from the vaginal microflora such as *L. crispatus* or *L. gasseri*.

So, we think that the content of the manuscript isn’t innovative enough to be of interest for the readers.

Our work combined optimization parameters identified from multiple source and report transformability on par with more commonly used species. This can serve as a foundation for other endeavors of that type and enable the genetic manipulation of the most widely distributed species across vaginal microflora worldwide.

Reviewer #3: The study is not novel but still provides some useful information. However, there are many problems that need to be addressed.

Major comments:

1. The "Introduction" is basically too long and didn't focus on the topic that the authors are talking about. The status of general plasmids/transformation in lactobacilli should be briefly discussed and mention the difficulty in L. jansenii.

We certainly agree with the reviewer and we apologize for that. We have now completely revised the introduction to make it much shorter and to the point.

2. the transformation can be strain-dependent, one strain is anyhow unconvincing and can not conclude an optimized electroporation for L. jansenii. The authors should try several different L. jansenii strains to validate the method.

The same issue is raised by reviewer 2. To answer this, we have obtained 3 independent *L. jensenii* from a bank established in a local hospital (Arnaud de Villeneuve UMR IRD224-CNRS5290-UM). We tested our protocol on these additional strains and added the results in Figure 4. We found that the protocol does permit to increase the transformability of all strain tested relative to the original procedure, though to various extent (10 to 40 fold increase).

3. the authors are highly recommended to try another replicon, pSH71 based plasmids, such as pNZ8148.

We would like to thank the reviewer for this suggestion. In fact, we used vector pTRK892 which precisely carries a pSH71 origin of replication. pSH71 is itself a derivative of pWVO1, a low copy origin of replication that is functional in both gram negative and positive bacteria. We have amended our manuscript to clarify all this.

Minor comments:

P3: "In turn, the characterization...of these gram-positive bacteria". The authors should track the recent publications. One of the major factors that influence the transformation efficiency is the restriction-modification system, please refer to Zuo et al., 2019[doi.org/10.1021/acssynbio.9b00114](http://doi.org/10.1021/acssynbio.9b00114), and Zuo et al., 2020 [doi.org/10.1016/j.biotechadv.2020.107654](http://doi.org/10.1016/j.biotechadv.2020.107654)

We have added a sentence in the conclusion to mention this as a perspective. We thank the reviewer for pointing out these interesting papers and have included them as references in the revised manuscript.

P13: table 1: it is strange that pTRKH2 and pTRKH3-slp-GFP share the same replicon and antibiotic-resistant gene, but with different copy numbers and transformation outcomes. The authors should carefully check the difference between these two plasmids and interpret the results.

We thank the reviewer for highlighting this discrepancy, which prompted us to fully sequence these two plasmids. Our result show that the replication origin of our version of pTRKH3-slp-GFP contains a deletion. We have contacted Addgene to report this problem. We have now removed pTRKH3-slp-GFP from our manuscript. As it was a direct derivative of pTRKH2, which is used throughout the manuscript, we think the loss of information due to this redaction is minimal.
