# Supplementary material for: An optimized electrotransformation protocol for Lactobacillus jensenii
Source: PLoS One. 2023 Feb 17;18(2):e0280935. doi: 10.1371/journal.pone.0280935 (PMC9937494; doi:10.1371/journal.pone.0280935)
Supplement: Attachment — Submitted filename: response to reviewers - 2.docx [file pone.0280935.s008.docx]

Reviewer #2: Major comments

Most of the previous comments has been properly addressed by the authors and we noticed the inclusion of three new strains of L. jensenii in this study.

Nevertheless, the results are improperly reported and thus the conclusions are not appropriate. The authors must correct two main faults:

1) Data are shown only by graphs. The original numerical data and their averages should be reported, at least as supplementary information.

Raw datas are now fully available in table 2 from *supplementary data.*

2) Trials have been performed in biological and technical replicates, but no statistical analysis is reported (neither in methods nor in results). Therefore, no statement about the significance of the observations is acceptable.

We added a Wilcoxon analysis on our graphs to support our observations in the results section. P-values data are available in table 3 from *supplementary data*.

Minor comments

Please check these possible mistakes (Raws are referred to the pdf version, disregarding blank lines):

P2 raws 5-6

low abundances = low abundance?

P2 raw 10

capacities = capacity?

P3 raw 11

to secreting = to secrete?

P3 raw 18

medium sized = medium-sized?

P4 raw 16

Cultures were incubating = Cultures were incubated?

P4 raws 22-23

pTRKH2 (kind gift from Michela Lizier, Addgene plasmid # 27168) = This reference is that of pTRKH3slpGFP from the previous version . pTRKH2 is not from Lizier’s lab

P5 raw 18

is as follow = is as follows?

P10 raw 2

different DNA quantity = different DNA quantities?

P11 raw 20

a few dozens = a few dozen?

P12 raw 4

any particular strains = any particular strain?

P12 raw 15

improve on our protocol = improve our protocol?

P13 raw 23

with high-precision = with high precision?

P14 raw 2

provides a mean = provides a means

Correction were added to the manuscript

p 16 S1 table

The whole 16S gene is shorter than the size indicated for the amplicon obtained with primers pFOR 16S L. jensenii and pREV 16S L. jensenii – check the value

We thank the reviewer for highlighting this discrepancy, we rechecked the sequence between our two primers and indeed the correct amplicon size is 1276bp.
